# Extracellular vesicles from senescent mesenchymal stromal cells are defective and cannot prevent osteoarthritis

**DOI:** 10.1186/s12951-024-02509-1

**Published:** 2024-05-16

**Authors:** Jérémy Boulestreau, Marie Maumus, Giuliana M. Bertolino, Karine Toupet, Christian Jorgensen, Daniéle Noël

**Affiliations:** 1 IRMB, University of Montpellier, INSERM U1183, Hôpital Saint-Eloi, 80 Avenue Augustin Fliche, Montpellier Cedex 5, 34295 France; 2 Clinical Immunology and Osteoarticular Disease Therapeutic Unit, Department of Rheumatology, CHU Montpellier, Montpellier, France

**Keywords:** Mesenchymal stromal cell, Extracellular vesicle, Senescence, Aging, Osteoarthritis, Regenerative medicine

## Abstract

Age is the most important risk factor in degenerative diseases such as osteoarthritis (OA), which is associated with the accumulation of senescent cells in the joints. Here, we aimed to assess the impact of senescence on the therapeutic properties of extracellular vesicles (EVs) from human fat mesenchymal stromal cells (ASCs) in OA. We generated a model of DNA damage-induced senescence in ASCs using etoposide and characterized EVs isolated from their conditioned medium (CM). Senescent ASCs (S-ASCs) produced 3-fold more EVs (S-EVs) with a slightly bigger size and that contain 2-fold less total RNA. Coculture experiments showed that S-ASCs were as efficient as healthy ASCs (H-ASCs) in improving the phenotype of OA chondrocytes cultured in resting conditions but were defective when chondrocytes were proliferating. S-EVs were also impaired in their capacity to polarize synovial macrophages towards an anti-inflammatory phenotype. A differential protein cargo mainly related to inflammation and senescence was detected in S-EVs and H-EVs. Using the collagenase-induced OA model, we found that contrary to H-EVs, S-EVs could not protect mice from cartilage damage and joint calcifications, and were less efficient in protecting subchondral bone degradation. In addition, S-EVs induced a pro-catabolic and pro-inflammatory gene signature in the joints of mice shortly after injection, while H-EVs decreased hypertrophic, catabolic and inflammatory pathways. In conclusion, S-EVs are functionally impaired and cannot protect mice from developing OA.

**Supplementary Information:**

The online version contains supplementary material available at 10.1186/s12951-024-02509-1.

## Introduction

Osteoarthritis (OA) is the most prevalent arthritic disease, characterized by cartilage degradation, subchondral bone sclerosis, osteophyte formation, synovial inflammation, and calcification of ligaments. The primary risk factors encompass obesity, genetic predisposition, and joint injuries resulting from traumas, with age emerging as the most predominant factor. This degenerative and debilitating disease affects over half of individuals aged 65 years and older, which yields around 30 million adults in the US population [1]. It is estimated that the number of people affected will double in the coming years, making OA a major socio-economic problem in industrialized societies [2]. Senescence is one of the hallmarks of aging that has been implicated in the pathogenesis and progression of most aging-associated diseases, including OA [3]. However, the exact mechanism connecting senescence and OA pathology remains unclear. Senescent cells are cell cycle-arrested and release a secretome containing various bioactive molecules, called the senescence-associated secretory phenotype (SASP). These molecules can be released as single molecules or within extracellular vesicles (EVs) and contribute to the pathogenesis of OA and the degradation of joint tissues. Targeting senescent cells therefore represents a novel approach to treating OA [4].

Among the therapeutic strategies being investigated for the treatment of OA, mesenchymal stromal cells (MSCs) represent a promising approach. A large number of studies have shown that MSCs can improve the altered phenotype of OA chondrocytes in vitro and have tissue-supporting functions in vivo by reducing inflammation and protecting cartilage and bone from degradation through the production of trophic factors [5–8]. In clinics, a meta-analysis of available data indicated that MSC-based treatment improves pain and functional parameters [9]. More recently, EVs isolated from MSCs were reported to reproduce most of the therapeutic effects of parental MSCs. They improved the dysregulated phenotype of OA chondrocytes in vitro and exerted a therapeutic effect in murine models of OA or cartilage repair [10–13]. However, in the perspective of autologous therapies for OA and other degenerative diseases, MSCs will be isolated from aged patients, which raises the question on their senescence status and function preservation. Indeed, it has been reported that the pool of stem cells decreases with age [14]. It has also been shown that EVs from aged or senescent MSCs may lose their regenerative potential and negatively influence the function of recipient cells through the release of SASP factors (for review, see [15]). However, to date, no study has evaluated the therapeutic effect of EVs from senescent MSCs in OA. The objective of the present study was to investigate the functional role of EVs isolated from senescent adipose tissue-derived MSCs (ASCs), in vitro on OA human chondrocytes and synoviocytes and, in vivo in a preclinical murine model of OA.

## Materials and methods

### Cells and culture media

The study was approved for specimen recovery by the French Ministry of Research and Innovation and the Personal Data Protection Ethics Committee (CPP) of Languedoc-Roussillon (approval DC-2010-1185). ASCs were obtained from 7 healthy donors (aged 48 ± 5 years), from surgical residues obtained after aesthetic liposuction. Isolation and characterization of ASCs have been performed as reported previously and expanded in α-MEM medium containing 10% fetal calf serum (FCS), 100 µg/mL penicillin/streptomycin (PS), 2 mM glutamine (Glu), and 1 ng/mL basic fibroblast growth factor (CellGenix, Freiburg, Germany) [16]. ASCs were used in passage 2.

Chondrocytes were isolated from the femoral condyles of patients with OA undergoing total knee replacement surgery (mean age: 74 ± 2 years). Briefly, cartilage slices were incubated in 2.5 mg/mL pronase (Sigma-Aldrich, Saint-Quentin-Fallavier) at 37 °C for 1 h followed by 2 mg/mL collagenase type II (Sigma-Aldrich), at 37 °C overnight. Cell suspensions were then filtrated through cell strainer filters with 70 μm pores and cultured in DMEM/PS/Glu/10% FCS (proliferative medium) at the density of 25,000 cells/cm² till the end of passage 0.

Synoviocytes were isolated from synovial tissue from patients with OA undergoing total knee replacement surgery (mean age: 68 ± 6 years). Synovial tissue was fragmented and incubated with collagenase II (2 mg/mL) at 37^o^C for 2 h. Digested tissue was passed through cell strainers and the flowthrough was analyzed by cellular viability and number of cells. Cells were resuspended in FCS supplemented with 10% of DMSO and frozen before any expansion. For analysis of fibroblast-like synoviocytes, total synoviocytes were thawed and expanded in DMEM supplemented with 10% FCS, 100 µg/mL PS and 2 mM Glu until passage 4, before being used in experiments.

For coculture assays, chondrocytes (500,000 cells/well) were plated on the bottom of 6-well plates and treated with IL1β (10 ng/mL) for 48 h. Different doses of EVs (100, 500 and 2500 ng) were then added on top of chondrocytes and cultured in 3 mL of minimal medium (DMEM supplemented with PS, 0.35 mmol/L proline, 0.17 mmol/L ascorbic acid and 1 mmol/L sodium pyruvate) for 7 days or in proliferative medium containing 10 ng/mL IL1β for 2 days. Synoviocytes were thawed and plated on 6-well plates (500,000 cells/well) and treated with LPS (100 ng/mL) or a combination of IFNγ (20 ng/mL) and TNFα (10 ng/mL) for 24 h. Thereafter, 500 ng of EVs was added in 2 ml of minimal medium for 24 h before cells were analyzed.

### Model of etoposide-induced senescence

ASCs were seeded in proliferative medium in 6-wells or 24-well plates at the density of 2,000 cells/cm² for control cells and 6,000 cells/cm² for etoposide (ETO) treatment. ETO (25 µM) was added for 24 h and ASCs were then rinsed three times with PBS before fresh medium addition for 7 or 12 days.

### Cell proliferation assay

ASCs were trypsinized and the number of live cells was counted using Trypan Blue exclusion dye. Proliferation was assessed using the Cell Proliferation Elisa BrdU assay as described by the manufacturer (Roche SAS, Boulogne-Billancourt).

### Senescence-associated β-galactosidase

ASCs were fixed with 2.5% glutaraldehyde at room temperature (RT) for 5 min. After three washes with PBS, cells were incubated with a solution of 5 mM Potassium ferrocyanide, 5 mM Potassium ferricyanide, 200 mM citric acid/sodium phosphate buffer pH 6, 150 mM sodium Chloride, 2 mM magnesium chloride, 1 mg/mL of X-gal (Promega, Charbonnières-les-Bains, France) at 37 °C for 6 h. Cells were then examined and photographed under a microscope (EVOS M5000, Invitrogen, Illkirch). SA-β-Gal^+^ ASCs were quantified with the ImageJ software using the cell counter complement. Additionally, SA-β-Gal activity was assessed using the 96-Well Cellular Senescence Activity Assay following the manufacturer’s instructions (Cell Biolabs, Clinisciences, Nanterre). Fluorescence was measured using a Varioskan Flash microplate reader (ThermoFisher Scientific, Illkirch-Graffenstaden).

### Immunofluorescence assays

ASCs were fixed with 4% formaldehyde at RT for 15 min and permeabilized with 0.3% Triton X-100 in PBS for 1 h. For γH2AX staining, 5% normal goat serum was added during the permeabilization step. After three washes with PBS, ASCs were incubated with Phospho-histone γH2AX (Ser139) antibody (Cell Signalling Technology, Ozyme, St-Cyr-l’Ecole) at 4 °C overnight. Cells were then washed three times and incubated with Alexa Fluor 594 goat anti-rabbit IgG (H + L) (ThermoFisher Scientific) at RT for 2 h and DAPI for 10 min. Finally, cells were photographed under a microscope (EVOS M5000, Invitrogen). The γH2AX foci and nucleus area were quantified using the Image J software and the cell counter complement.

For F-actin staining, ASCs were incubated with 50 µg/mL fluorescent phalloidin conjugated with Tetramethyl-rhodamine B isothiocyanate (TRITC) in PBS at RT for 40 min. Cells were then stained with DAPI for 10 min and washed three times with PBS before being photographed. Using the Image J software, the cell surface and the corrected total cell fluorescence (CTCF) were quantified by the formula: CTCF = Integrated Density – (Area of selected cell x Mean fluorescence of background readings).

### RNA extraction and RT-qPCR

Total RNA was extracted from cells using 350 µL RLT buffer from the RNeasy Mini Kit according to the supplier’s recommendations (Qiagen, Les Ulis). Reverse transcription of 500 ng RNA was obtained by M-MLV reverse transcriptase (ThermoFisher Scientific). Real-time PCR was done on 10 ng cDNA using SYBR Green I Master mix (Roche Diagnostics, Meylan) and specific primers (Table 1).

**Table 1:** List of primers for PCR

Gene	Sequence forward	Sequence reverse
ACAN	TCGAGGACAGCGAGGCC	TCGAGGGTGTAGCGTGTAGAGA
COL2A1ΔB	CAGACGCTGGTGCTGCT	TCCTGGTTGCCGGACAT
COL1A1	CCTGGATGCCATCAAAGTCT	CGCCATACTCGAACTGGAAT
COL3A1	CGCCCTCCTAATGGTCAAGG	AGGGCCTGAAGGACCAGCTT
MMP3	TTGCGCCAAAAGTGCCTGTCT	GTACCCACGGAACCTGTCCCTC
MMP13	TAAGGAGCATGGCGACTTCT	GTCTGGCGTTTTTGGATGTT
IL6	AGACAGCCACTCACCTCTTCAG	TTCTGCCAGTGCCTCTTTGCTG
IL8	GAGAGTGATTGAGAGTGGACCAC	CACAACCCTCTGCACCCAGTTT
p14ARF	CCCTCGTGCTGATGCTACTG	ACCTGGTCTTCTAGG AAGCGG
p15INK4b	GACCGGGAATAACCTTCCAT	CACCAGGTCCAGTCAAGGAT
p16INK4a	GAAGGTCCCTCAGACATCCCC	CCCTGTAGGACCTTCGGTGAC
p21cdkn1a	AGGTGGACCTGGAGACTCTCAG	TCCTCTTGGAGAAGATCAGCCG
p27KIP1	ATAAGGAAGCGACCTGCAACCG	TTCTTGGGCGTCTGCTCCACAG
p57KIP2	GCGGCGATCAAGAAGCTGT	GCTTGGCGAAGAAATCGGAGA
RPS9	GATTACATCCTGGGCCTGAA	ATGAAGGACGGGATGTTCAC

Total RNA was extracted from joint tissues after mechanical dissociation of joints previously stored in Trizol using Ultra-Turrax. Chloroform (200 µL) was then added to the suspension of crushed tissue and incubated at RT for 3 min before centrifugation at 12,000 ×g, at 4 °C for 15 min. The aqueous phase was recovered and mixed with 600 µL of 70% ethanol. The suspension was then loaded onto columns of the RNeasy Mini Kit according to the supplier’s recommendations (Qiagen, Les Ulis).

Values were normalized to the Ribosomal Protein S9 (RPS9) housekeeping gene and expressed as a relative expression or fold change using the respective formulae 2^−ΔCT^ or 2^−ΔΔCT^.

### Protein detection and ELISA assays

Cells and EV pellets were resuspended with RIPA buffer (Sigma) and incubated for 20 min on ice for protein extraction. After protein quantification by Micro BCA Protein Assay Kit (ThermoFisher Scientific), protein preparations were analysed by Western Blot and ELISA.

For Western blot analysis, 20 µg of proteins were mixed with lithium dodecyl sulfate (LDS) and reducing buffer to a final volume of 40 µL, and incubated at 70^o^C for 10 min. The protein preparation was loaded and separated in Novex 4 to 20%, Tris-Glycine polyacrylamide gels (Thermo Fischer Scientific), and then transferred to nitrocellulose membrane using the iBlot2 system (Life Technologies). The transferred membrane was then blocked with Tris-buffered saline, 0.1% Tween 20 (TBS-T) with 5% milk, incubated with primary antibodies for LOXL4 detection (ab88186, 1:2000, Abcam) and β-actin (AC-15, 1:5000, Sigma), and then the secondary anti-rabbit (#7074, 1:2000, Cell Signaling Technology) and anti-mouse (A9044, 1:100000, Sigma) antibodies. The membrane was treated with Sirius HPR substrate (Advansta, San Jose, USA) and analysed with the ChemiDoc (BioRad, Hercules, USA) imaging system.

Supernatants from cells were recovered and stored at -20 °C until use. Quantification of MMP-3, MMP-13, IL-6, IL8, HGF and VEGF was performed using specific Enzyme-Linked Immunosorbent Assays (Bio-Techne, RnD Systems, Rennes). ICAM-1 (Abcam, Cambridge, UK), GPC1 (RnD Systems), APOE (Abcam), COL15A (Novus Biologicals, Littleton, USA) were detected in cell supernatant, cell lysate (1 µg) and/or EV lysate (5 µg) using ELISA kits as per manufacture recommendation.

### Flow cytometry for marker detection on synoviocytes

Synoviocytes were gently detached from cell culture plates by incubation with Versene (Gibco) for 45 min at 37^o^C before being stained with REAfinity antibodies anti-human CD45, CD73, CD11b, CD86, CD80 and CD163 (Miltenyi) at RT for 15 min and washed in PBS containing 0.5% BSA and 2mM EDTA, as per manufacturer’s recommendation. Cells were analyzed using the FACS Canto II Cytometer (BD).

### EV Production, isolation and characterization

Production of conditioned medium (CM) and isolation of EVs were performed as previously described [17]. Briefly, ASCs were cultured in α-MEM medium containing 3% EV-depleted FCS for three days. Total EVs were recovered from the conditioned medium by differential ultracentrifugation (last step at 100,000×g, 4 °C for 2 h). EVs were characterized as recommended by the International Society of Extracellular Vesicles (ISEV) [18]. EV size and concentration were determined by Nanoparticle Tracking Analysis, as described [17]. The structure was observed using cryo-TEM while the total RNA and protein contents were determined using the RNeasy Micro Kit (Qiagen, Les Ulis) and the Micro BCA Protein Assay Kit (ThermoFisher Scientific), respectively. Surface marker expression was analyzed using fluorophore-conjugated antibodies. EVs (1 µg of equivalent proteins) were coated onto 4 μm aldehyde/sulfate latex beads by incubation at 4 °C overnight. Beads coated with EVs were then washed 3 times in PBS and incubated with specific antibodies for CD11b, CD29, CD44, CD45, CD63, CD81, CD90 and HLA-ABC (BD Biosciences) by flow cytometry. EVs were used freshly prepared.

### Mass spectrometry and analysis

EVs (2 µg) were lysed and total protein content was reduced, alkylated and digested with trypsin. The peptides were then desalted and injected on a nanoLC-Q-TOF Impact II (Brüker). Each sample was injected in triplicate.

Protein identification was performed with Maxquant software (version 1.6.17.0). The parameters used were the following: the digestion enzyme is trypsin, the number of missed cleavages is 1, a mass tolerance of 10 ppm for parent ions and 0.05 Da for MS/MS spectra was used, the minimum peptide size is 5 amino acids, the maximum peptide mass is 4600 Da and a protein identification FDR was set at 2.5%. The Uniprot database of the human proteome was used as reference (version 01/02/2021). Some modifications, induced by the sample preparation protocol, were studied: asparagine deamidation and methionine oxidation as variable modifications and cysteine carbamidomethylation as fixed modification. The maximum number of modifications for a peptide was 5.

LFQ intensities for each protein were normalized by the initial protein amount before data processing, performed with the LFQ-Analyst platform. Proteins considered as contaminants and redundant were removed. For 3 biological replicates, proteins without at least 2 valid values for at least one group were eliminated. The LFQ data for each protein were transformed by applying the log2(x) formula and grouped according to donors and conditions (4 donors: D1, D2, D3, D4 and 4 conditions: Control, 2DG, TI, Oligo). The data was then normalized to meet a normal distribution and missing values were imputed. Protein-wise linear models combined with empirical Bayes statistics were used for the differential expression analyses. The *limma* package from R Bioconductor was used to generate a list of differentially expressed proteins for each pair-wise comparison. A cutoff of the adjusted p-value of 0.05 (t-statistic correction) along with a |log2 fold change| of 0.5 has been applied to determine significantly regulated proteins in each pairwise comparison.

### Collagenase-induced osteoarthritis model and joint imaging

The collagenase-induced OA (CIOA) murine model was performed as previously described [19] and following guidelines and regulations of the Ethical Committee for animal experimentation of the Languedoc-Roussillon (Approval APAFIS#5349-2016050918198875). Briefly, 5 µL of 1Unit type VII collagenase or saline (control group; CT) were administered intra-articularly (IA) in the knee joint of 10 weeks-old C57BL/6 mice at days 0 and 2. At day 7, collagenase-injected groups of 22 mice received IA injections of EVs (250 ng/5 µL) or 5 µL saline (CIOA). Part of the mice (*n* = 7/group) were euthanatized on day 14 and knee joints were recovered. Soft tissues around the joints were carefully removed, and the joints were stored in 1 mL Trizol at -80 °C.

On day 42, paws were recovered from the other part of the mice (*n* = 15) and fixed in 4% formaldehyde. For bone analysis, hind paws were scanned in a Micro-Computed Tomography (µCT) scanner (SkyScan 1176, Bruker, Kontich, Belgium) and 3D image stacks were reconstructed using the NRecon software (Bruker). The quantification of the subchondral bone of the tibia and calcification of the meniscus and ligaments was performed using the CTAn software (Bruker). Reconstructed 3D images of joints were obtained using Avizo software (Avizo Lite 9.3.0, FEI, France). For cartilage analysis, tibia plateaus were scanned with a confocal laser scanning microscope (CLSM; TCS SP8, Leica Microsystems, Nanterre, France). Stacks of images were analyzed to quantify several cartilage morphometric parameters using the Avizo software (FEI Visualization Sciences Group, Lyon).

### Histological analysis

After µCT and CLSM analyses, hind paws were decalcified using 5% formic acid at RT for 2 weeks and then embedded in paraffin. Frontal sections of tibias were cut (3 slices of 7 μm each 100 μm; first section at 50 μm below the cartilage surface) and stained with safranin O and fast green. Cartilage degradation was quantified using the modified Pritzker OARSI score.

### Statistical analysis

Statistical analyses were performed using the GraphPad 9 Prism Software. Data distribution was assessed using the Shapiro–Wilk normality test. The statistical analyses are indicated in the figure legends. Data are presented as mean ± SEM. * *p* < 0.05; ** *p* < 0.01; *** *p* < 0.001; ****< 0.0001.

## Results

### Generation of DNA damage-induced senescence in ASCs

**Fig. 1:**
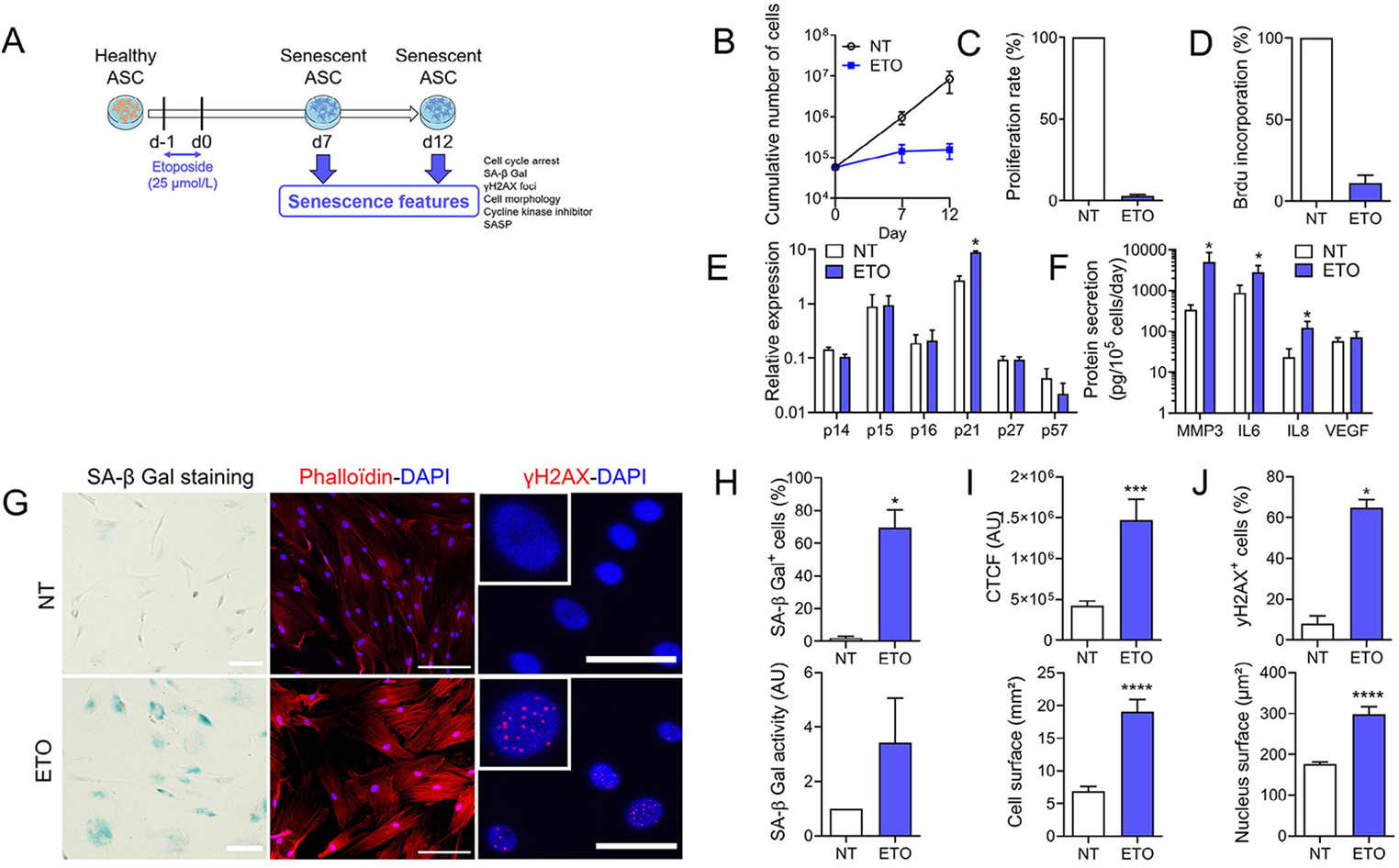
Etoposide-induced senescence in human ASCs. (**A**) Schematic protocol for etoposide-induced senescence in ASCs. (**B**) Cumulative number of ASCs in non-treated (NT) and etoposide (ETO)-treated ASCs (*n* = 3). (**C**) Proliferation rate of NT and ETO ASCs at day 12 (*n* = 3). (**D**) Percentage of BrdU incorporation in NT and ETO ASCs at day 12 (*n* = 3). (**E**) Relative expression of Cyclin-Dependent Kinase Inhibitors (p14ARF, p15INK4b, p16INK4a, p27KIP1 and p57KIP2) in NT or ETO ASCs at day 12 (*n* = 3). (**F**) Amounts of proteins secreted by NT and ETO ASCs quantified by ELISA (*n* = 6). (**G**) Representative pictures of SA-β-Galactosidase (SA-β-Gal in blue, bars: 100 μm) staining, phalloidin/DAPI (actin stress fibers in red and nuclei in blue; bars: 100 μm) and γH2AX/DAPI (γH2AX foci in red and nuclei in blue; bars: 50 μm) staining in NT and ETO ASCs. (**H**) Percentage of SA-β-Gal positive ASCs (upper panel) and SA-β-Gal activity quantified by fluorometry (lower panel) (*n* = 4). (**I**) Quantification of Corrected Total Cell Fluorescence (CTCF; upper panel) and cell surface (lower panel) stained with fluorescent Phalloidin (*n* = 9–14). (**J**) Percentage of ASCs with γH2AX positive foci in the nucleus (upper panel) and quantification of the nucleus surface stained with DAPI (*n* = 4–12). Data are shown as mean ± SEM. Statistical analysis used the Mann-Whitney test (**B**, **E**, **F**, **H**: upper panel, **I**, **J**) or the one-sample Wilcoxon test (**C**, **D**, **H**: lower panel). *p < 0.05, ***p < 0.001, ****p < 0.0001

We used etoposide (ETO) to generate a model of DNA damage-induced senescence (Fig. 1A). A 24 h treatment with 25 µmol/L ETO was sufficient to induce a proliferation arrest of ASCs as shown by the low cumulative cell number, proliferation rate and BrdU incorporation percentage at day 12 (Fig. 1B-D). This was associated with a significant increase of the cyclin-dependent kinase inhibitor (CDKI) p21cdkn1a, while the expression of other CDKIs (p14ARF, p15INK4b, p16INK4a, p27KIP1 and p57KIP2) was not changed (Fig. 1E). Secretion of several proteins of the SASP (MMP3, IL6 and IL8) was highly increased in ETO-treated ASCs compared to control ASCs (Fig. 1F). We also observed increased detection in the SA-β-Gal activity and a high percentage of ASCs stained for SA-β-Gal (Fig. 1G-H). The number of stress fibers stained with phalloidin and quantified by CTCF was increased as well as the cell surface of ETO-treated ASCs compared to untreated ASCs (Fig. 1G, I). Finally, ETO-induced DNA damage foci were visualized by γH2AX staining, and we observed an increased percentage of ASCs with γH2AX^+^ nuclei and larger nucleus surfaces (Fig. 1G, J). All these senescence-related features were observed both on days 7 and 12 (Fig. S1), indicating the non-reversible nature of the induced senescence and the possibility of analyzing senescence parameters on day 7. These data demonstrated that etoposide addition mimics a DNA damage-induced senescence model in ASCs.

### Senescent ASCs produce higher amounts of EVs

**Fig. 2:**
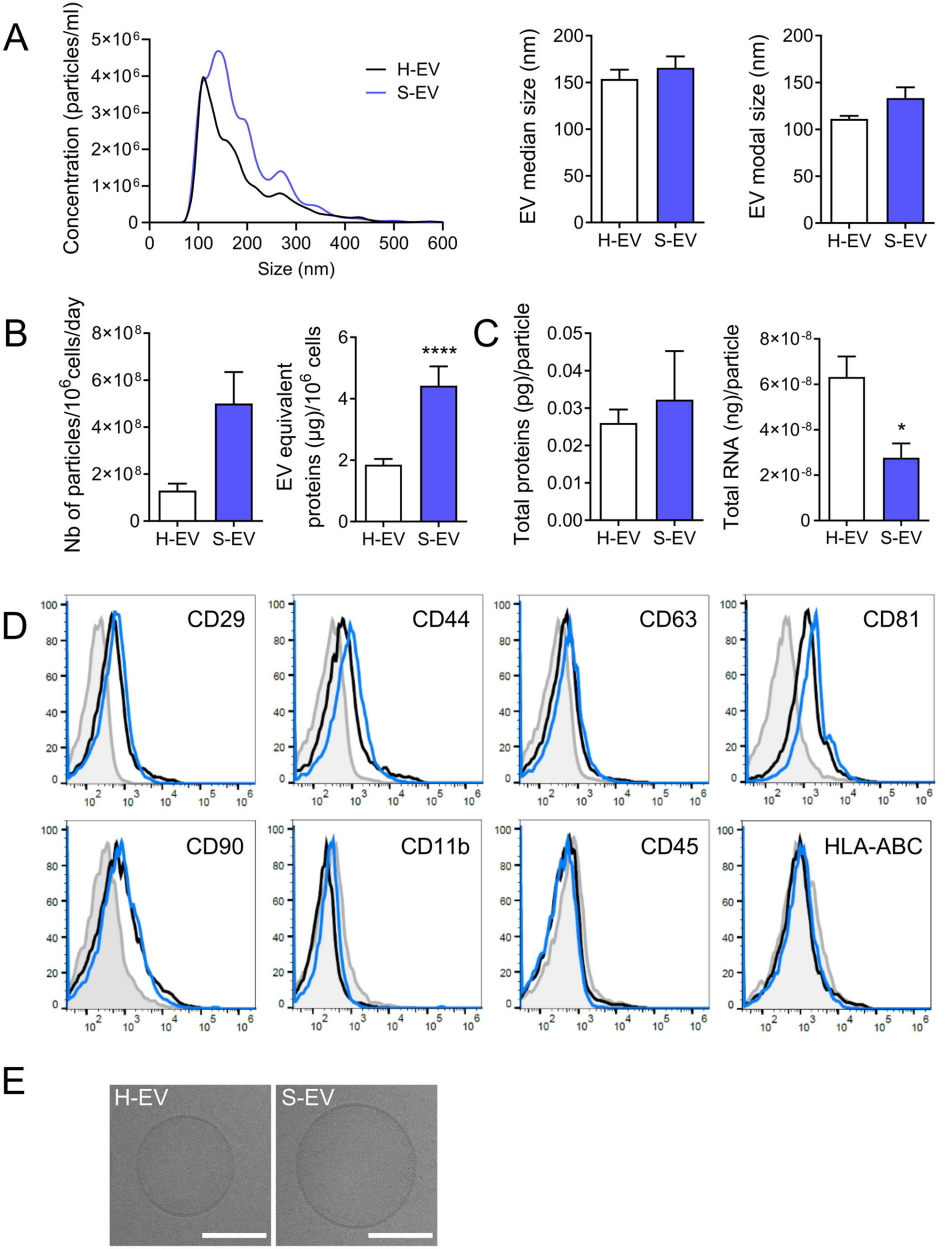
Characterization of extracellular vesicles isolated from human ASCs. (**A**) Size distribution of EVs isolated from healthy ASCs (H) or ETO-induced senescent ASCs (S) by Nano Tracking Analysis. The median and modal sizes of EVs are shown in the middle and right panels, respectively (*n* = 3). (**B**) EV numbers produced by 1 million ASCs per day (left panel) (*n* = 3) or EV equivalent in µg of proteins produced by 1 million ASCs (right panel) (*n* = 20–23). (**C**) Quantity of total proteins (left panel) and total RNA (right panel) produced per particle (*n* = 3). (**D**) Expression profile of membrane markers on the surface of H-EVs (black line), S-EVs (Blue line) or isotypic control (grey histogram) by FACS. (**E**) Representative pictures of H-EVs and S-EVs by cryo-transmission electron microscopy (scale bar: 100 nm). Data are shown as mean ± SEM. Statistical analysis used the Mann-Whitney test. *p < 0.05, ****p < 0.0001

We then isolated and characterized EVs from healthy and senescent ASCs. EVs from senescent ASCs (S-EVs) displayed a different size distribution and were slightly bigger than EVs from healthy ASCs (H-EVs) as shown by their median and modal sizes (Fig. 2A). The number of particles produced by S-ASCs was higher than that produced by H-ASCs, as well as the quantity of EV-equivalent total proteins (Fig. 2B). The cargo of H-EVs and S-EVs was similar in terms of total protein amount per particle, but S-EVs had 2.3-fold less total RNA content (Fig. 2C). Several membrane markers including CD29, CD44, CD63, CD81, CD90, were expressed at similar levels on S-EVs and H-EVs, while hematopoietic markers (CD11b, CD45) and HLA-ABC were not detected (Fig. 2D). The presence of a bilayer membrane in both H-EVs and S-EVs samples was confirmed by cryo-TEM imaging (Fig. 2E). All these data indicated that H-EVs and S-EVs exhibit slightly different characteristics.

### The therapeutic effect of senescent ASC-EVs on OA chondrocytes is lost when chondrocytes are cultured in proliferative conditions

**Fig. 3:**
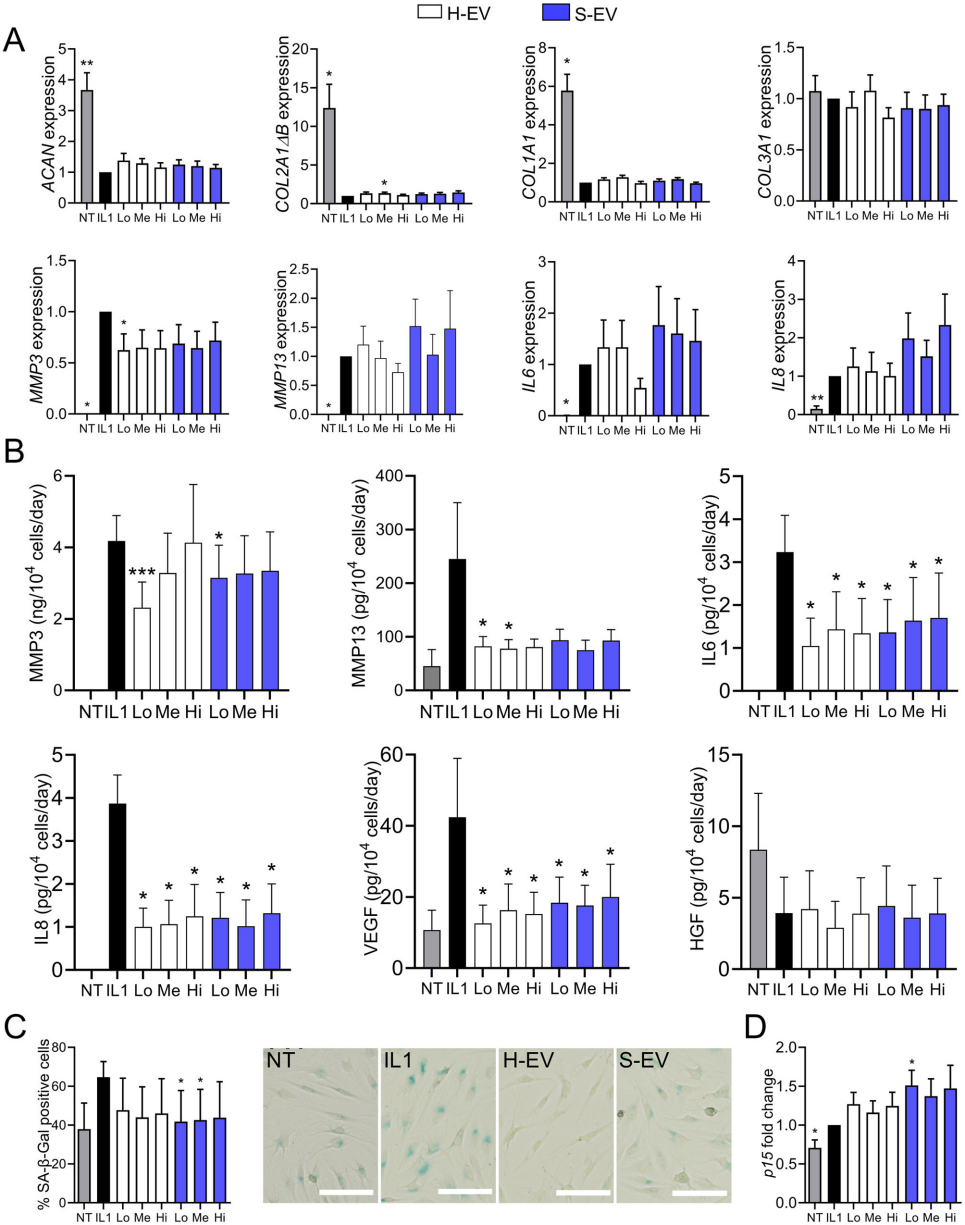
Senescent ASC-EVs exert a chondroprotective effect on OA chondrocytes cultured in resting conditions. Primary human chondrocytes were pretreated with 10 ng/mL IL1β (IL1) or not (NT) for 48 h. Different amounts (Low (Lo): 100 ng; Medium (Me): 500 ng; High (Hi): 2.5 µg) of EVs from healthy or senescent ASCs (H-EV or S-EV) were added for 7 days. (**A**) Expression of chondrocyte markers as expressed as fold change (*n* = 7). (**B**) Quantification of several factors in the culture supernatants by ELISA (*n* = 7). (**C**) Percentage of SA-β-Gal positive chondrocytes (left panel) and representative pictures of each group (right panel; H-EV and S-EV stand for medium EV dose for each type of EV) (*n* = 7). (**D**) Expression of the CDKI as expressed as fold change (*n* = 7). Data are shown as mean ± SEM. Statistical analysis used the one-sample Wilcoxon test (**A**, **D**) the t-test (**B**) or the Mann-Whitney test (**C**) comparing the treated sample to the IL1β control. *p < 0.05

We then evaluated the effect of S-EVs on the phenotype of OA chondrocytes cultured in resting conditions (minimal medium) to mimic the native cartilage environment as previously described [7]. The addition of both S-EVs and H-EVs slightly up-regulated the expression of the anabolic marker *COL2A1ΔB* and down-regulated *MMP3* at day 7, while the expression of other markers was not significantly modulated (Fig. 3A). However, at protein level, both H-EVs and S-EVs significantly down-regulated the catabolic markers MMP3, MMP13 as well as the inflammatory factors IL6, IL8 and the growth factor VEGF (Fig. 3B). Concomitantly, the number of chondrocytes that stained positive for SA-β-Gal was significantly lower with S-EVs while the expression of *p15* was increased (Fig. 3C-D). These data support the notion that S-EVs maintained the beneficial effect observed with H-EVs on OA chondrocytes.

**Fig. 4:**
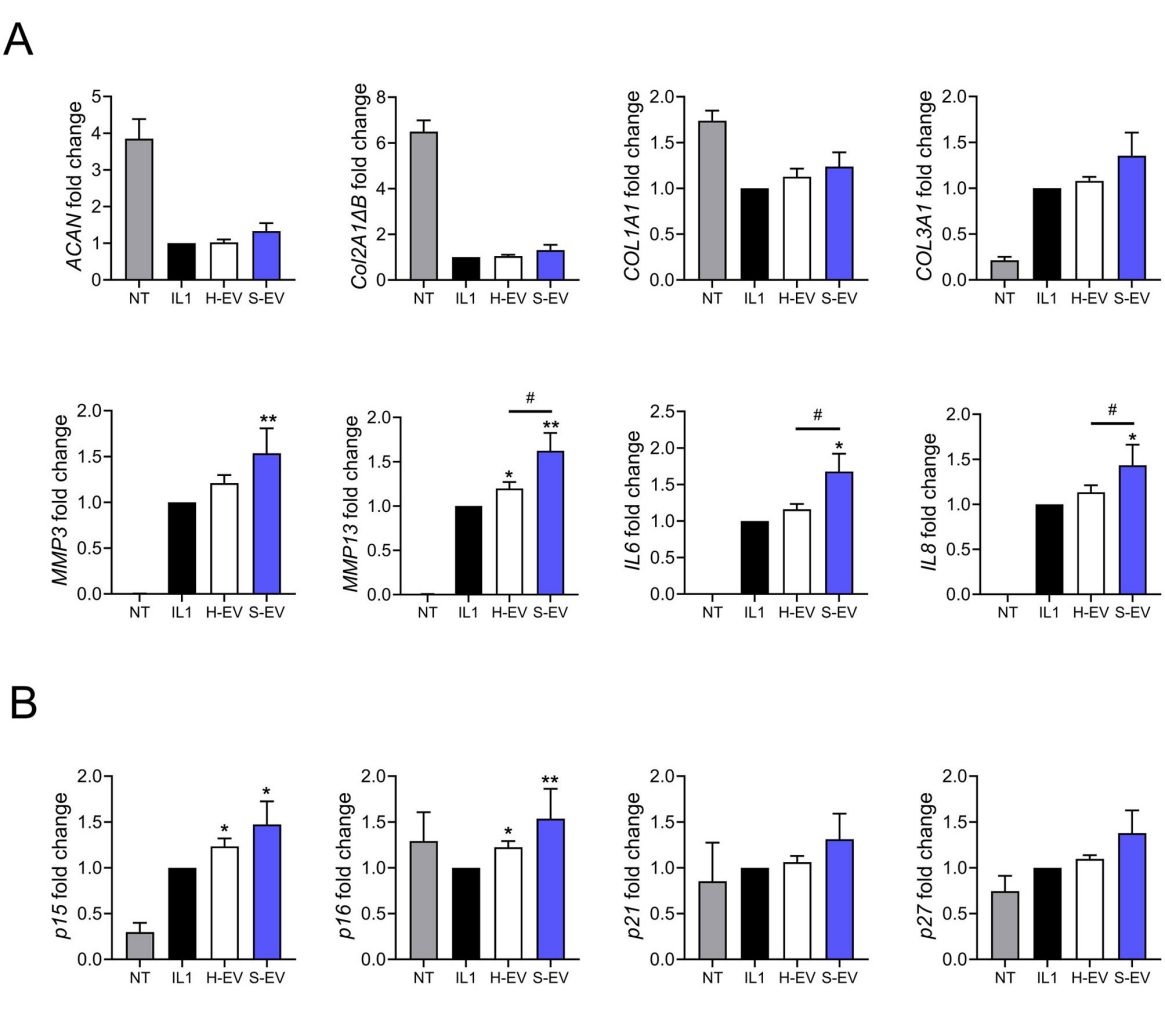
Senescent ASC-EVs lose their chondroprotective effect on OA chondrocytes when cultured in proliferating conditions. Primary human chondrocytes were pretreated with 10 ng/mL IL1β (IL1) or not (NT) for 24 h and EVs (Medium dose: 500 ng) from healthy or senescent ASCs (H-EV or S-EV) were added for 48 days. (**A**) Expression of chondrocyte markers as expressed as fold change (*n* = 10). (**B**) Expression of CDKIs as expressed as fold change (*n* = 10). Data are shown as mean ± SEM. Statistical analysis used the one-sample Wilcoxon test comparing the treated sample to the IL1β control (*p < 0.05, **p < 0.01) or the Mann-Whitney test for comparing H-EV and S-EV samples (# *p* < 0.05)

However, the chondroprotective effect of S-EVs was unexpected when compared to previous studies [10–13] and we hypothesized that the culture conditions might have influenced the response of chondrocytes to EVs. To investigate this hypothesis, we cultured chondrocytes in proliferative conditions (FBS-containing medium) during EV exposure. The addition of both types of EVs had no effect on the anabolic activity of chondrocytes as shown by no change in the expression of ACAN, *COL2A1ΔB, COL1A1, COL3A1*, while S-EVs significantly increased the catabolic and inflammatory markers *MMP3, MMP13, IL6, IL8* (Fig. 4A). In addition, S-EVs tended to increase the expression of CDKIs (Fig. 4B). At the protein level, secretion of the SASP factors was undetectable or did not change significantly (data not shown). Altogether, these results indicate that S-EVs upregulate several catabolic, inflammatory and senescence-associated markers that is not observed with H-EVs when chondrocytes are in proliferating conditions.

### Senescent ASC-EVs induce a pro-inflammatory phenotype in synovial macrophages and alter the phenotype of synovial fibroblasts

**Fig. 5:**
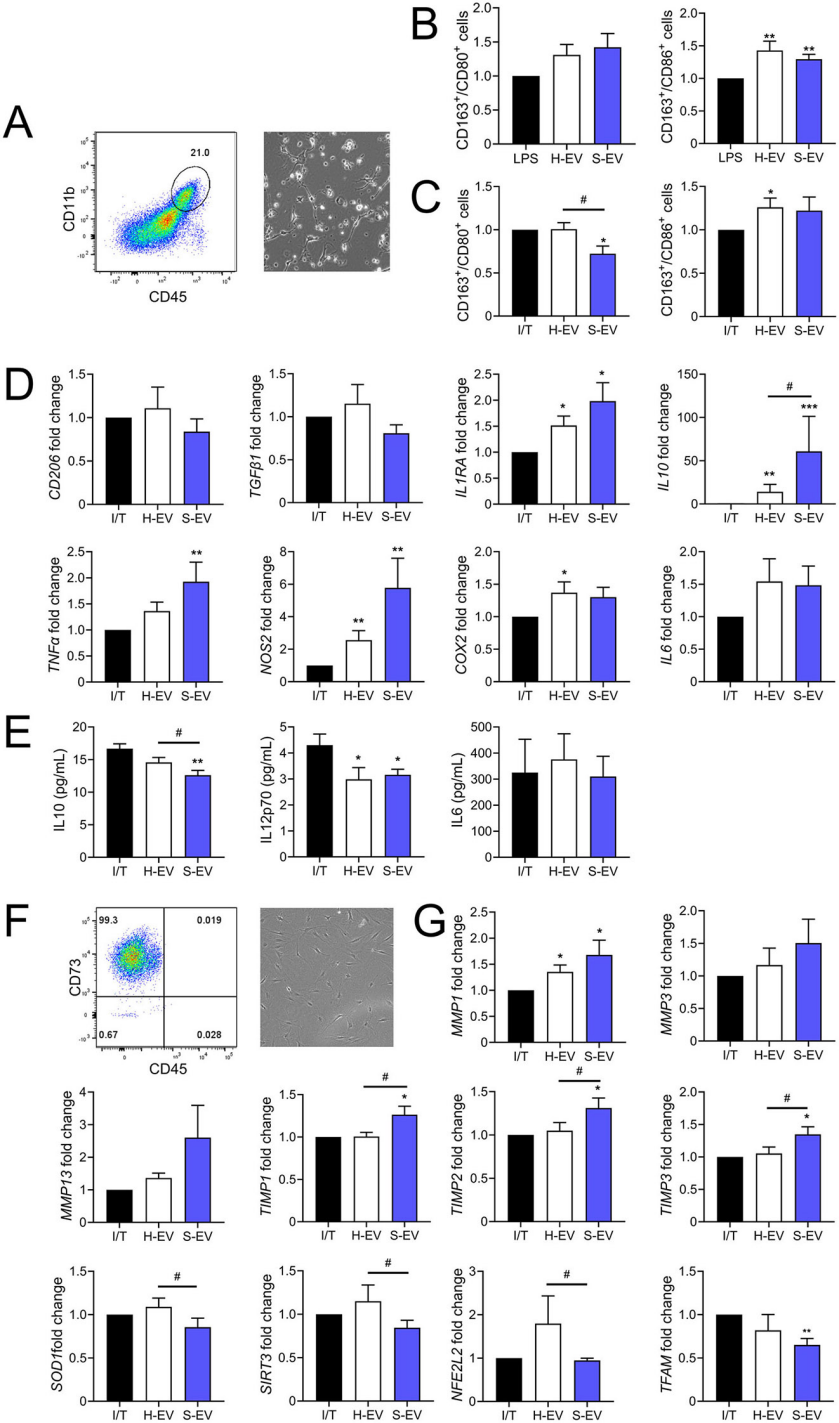
Senescent ASC-EVs induce a pro-inflammatory phenotype in synovial macrophages and alter the phenotype of synovial fibroblasts. Primary human synoviocytes were pretreated with LPS or IFNγ and TNFα (I/T; 20 ng/mL and 10 ng/mL, respectively) for 24 h and EVs (Medium dose: 500 ng) from healthy or senescent ASCs (H-EV or S-EV) were added for 48 days. (**A**) Representative gating strategy of macrophages (CD11b^+^ cells in the CD45^+^ cells) in the synovial cell population and picture of the synovial cells at P0. (**B**) The average ratio of CD163^+^/CD80^+^ and CD163^+^/CD86^+^ macrophages after LPS stimulation (*n* = 12). (**C**) The average ratio of CD163^+^/CD80^+^ and CD163^+^/CD86^+^ macrophages after I/T stimulation (*n* = 10). (**D**) Expression of macrophage markers as expressed as fold change (*n* = 10). (**E**) Quantification of cytokines in the supernatants of I/T-stimulated macrophages by ELISA (*n* = 8). (**F**) Representative gating strategy of synovial fibroblasts (CD73^+^ and CD45^−^ cells) in the synovial cell population and picture of the synovial cells at P4. (**G**) Expression of markers in fibroblasts as expressed as fold change (*n* = 9). Data are shown as mean ± SEM. Statistical analysis used the one-sample Wilcoxon test comparing the treated sample to the IL1β control (*p < 0.05, **p < 0.01) or the Mann-Whitney test for comparing H-EV and S-EV samples and in panel F (# *p* < 0.05)

We further wondered whether S-EVs might exert a deleterious impact on the other cells from the joint compartment. We recovered the synovial cell population from OA patients at P0. The percentage of CD11b^+^/CD45^+^ macrophages was 16.62 ± 4.65% while CD45^−^/CD73^+^ fibroblasts represented 45.23 ± 5.7% (Fig. 5A). After stimulation of the cells with LPS, an increased number of M2 anti-inflammatory macrophages expressing CD163 versus M1 macrophages expressing CD80 and CD86 was observed with both H-EVs and S-EVs (Fig. 5B). However, after stimulation with IFNγ and TNFα (common cytokines in the OA joint environment), the ratio was in favor of a pro-inflammatory phenotype (Fig. 5C) when cells were treated with S-EVs. At the RNA level, a trend toward a lower expression of the anti-inflammatory markers of macrophages *CD206* and *TGFβ1* was also noticed in synoviocytes exposed to S-EVs while both pro- and anti-inflammatory factors were increased (Fig. 5D). Nevertheless at the protein level, the secretion of IL10 was significantly decreased when S-EVs were added even though the secretion of IL12 and IL6 (markers of M1 macrophages) was similar with S-EVs and H-EVs, suggesting a shift toward an inflammatory phenotype after cells were treated with S-EVs (Fig. 5E).

In the synovial fibroblast population obtained at P4 (99.19 ± 0.37% CD45^−^/CD73^+^ cells) and treated with both EV types, the increase of metalloproteinases (*MMP1, MMP3, MMP13*) was concomitant with the increase of MMP inhibitors (*TIMP1, TIMP2, TIMP3*) (Fig. 5F-G). However, the anti-aging and anti-oxidative markers *SOD1, SIRT3, NFE2L2*, and *TFAM* were decreased in fibroblasts exposed to S-EVs. The results suggest that synovial macrophages and fibroblasts might up-regulate the anti-inflammatory and anti-catabolic markers to counterbalance the pro-aging signals released by S-EVs but the overall response might be in favor of a deleterious effect of S-EVs on the various cell types in the joint compartment.

### Differential cargo composition in S-EVs and H-EVs

**Fig. 6:**
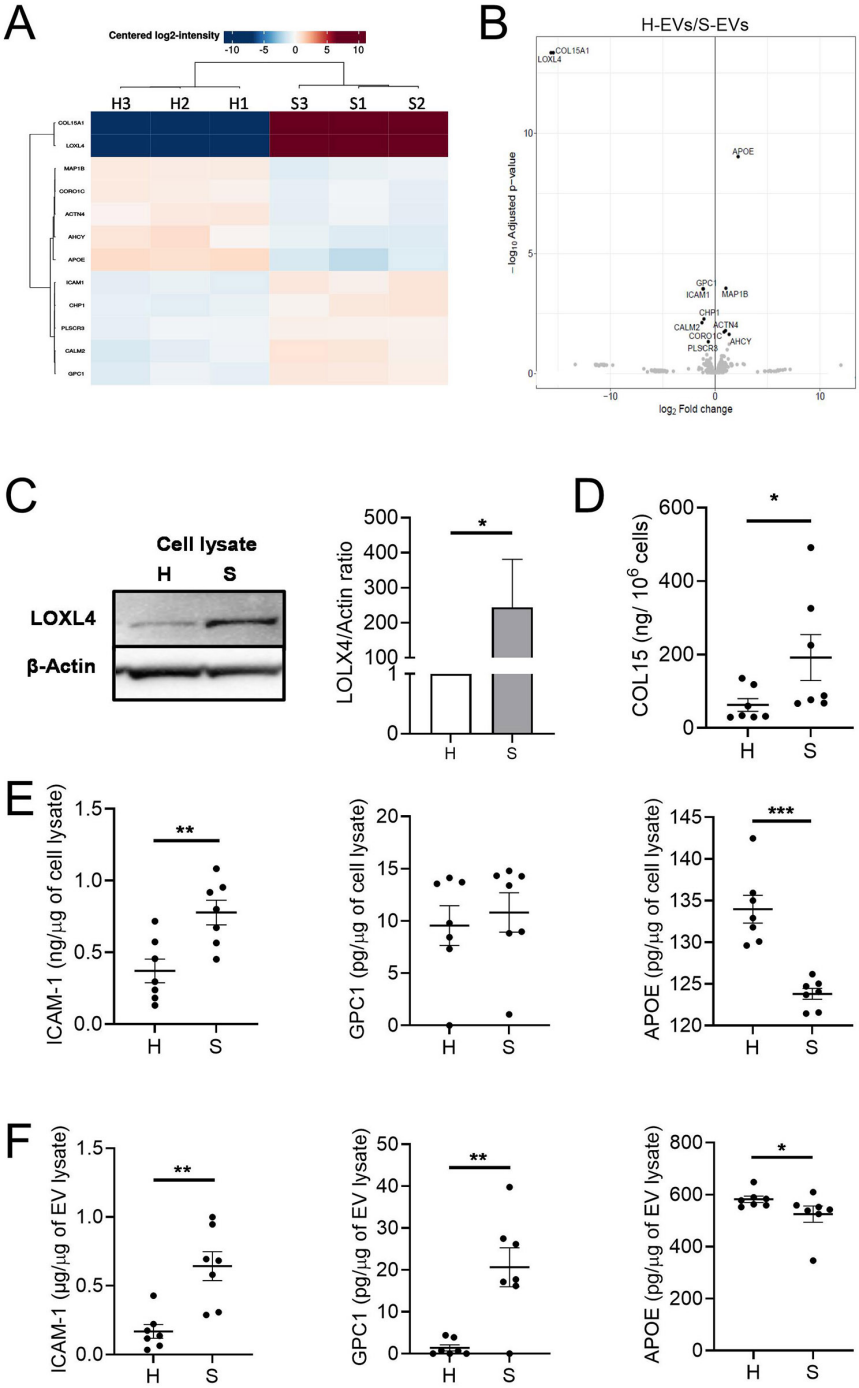
Comparative proteomic analysis reveals differential cargo composition in S-EVs and H-EVs. (**A**) Heatmap plot and (**B**) volcano plot of the differential protein contents obtained by mass spectrometry analysis of H-EVs and S-EVs. (**C**) Detection of LOXL4 on cell lysates by western blot (plot representative of three biological replicates). The graph represents the quantification of the LOXL4 band normalised by the β-actin band, which was used as loading control (*n* = 3). (**D**) Detection of COL15 in cell supernatants by ELISA (*n* = 7). (**E**) Detection of ICAM-1, GPC1 and APOE in cell lysates by ELISA (*n* = 7). (**F**) Detection of ICAM-1, GPC1 and APOE in EV lysates (*n* = 7). Data are shown as mean ± SEM. Statistical analysis used the Mann-Whitney test for comparing samples pairwise with *p < 0.05, **p < 0.01, ***p < 0.001

To understand the dysfunction of S-EVs compared to H-EVs, a comparative proteomic analysis was performed by mass spectrometry. Twelve proteins were found to be differentially expressed in S-EVs and H-EVs (Fig. 6A). Among the proteins that were the most significantly modulated in S-EVs, five proteins were selected to be confirmed by detection in protein-based assays: type XV collagen (COL15A), lysyl oxidase like-4 (LOXL4), apolipoprotein E (APOE), glypican-1 (GPC1), and intercellular adhesion molecule 1(ICAM-1) (Fig. 6B). The presence of these proteins was validated in cell supernatants or lysates from both H-ASCs and S-ASCs (Fig. 6C-E). COL15A was detected only in the cell supernatants, while LOXL4 and ICAM-1 were detected in cell lysates, and these three proteins were increased in S-ASCs samples. Instead, APOE levels were decreased in S-ASC lysates. Similar findings were observed in S-EVs and H-EVs, except for COL15A and LOXL4 which were not detected (Fig. 6F). We therefore validated the differential cargo for three of the five most abundant proteins in the lysates from S-EVs and H-EVs.

### S-EVs are less efficient than H-EVs to protect cartilage and bone from alterations in OA

**Fig. 7:**
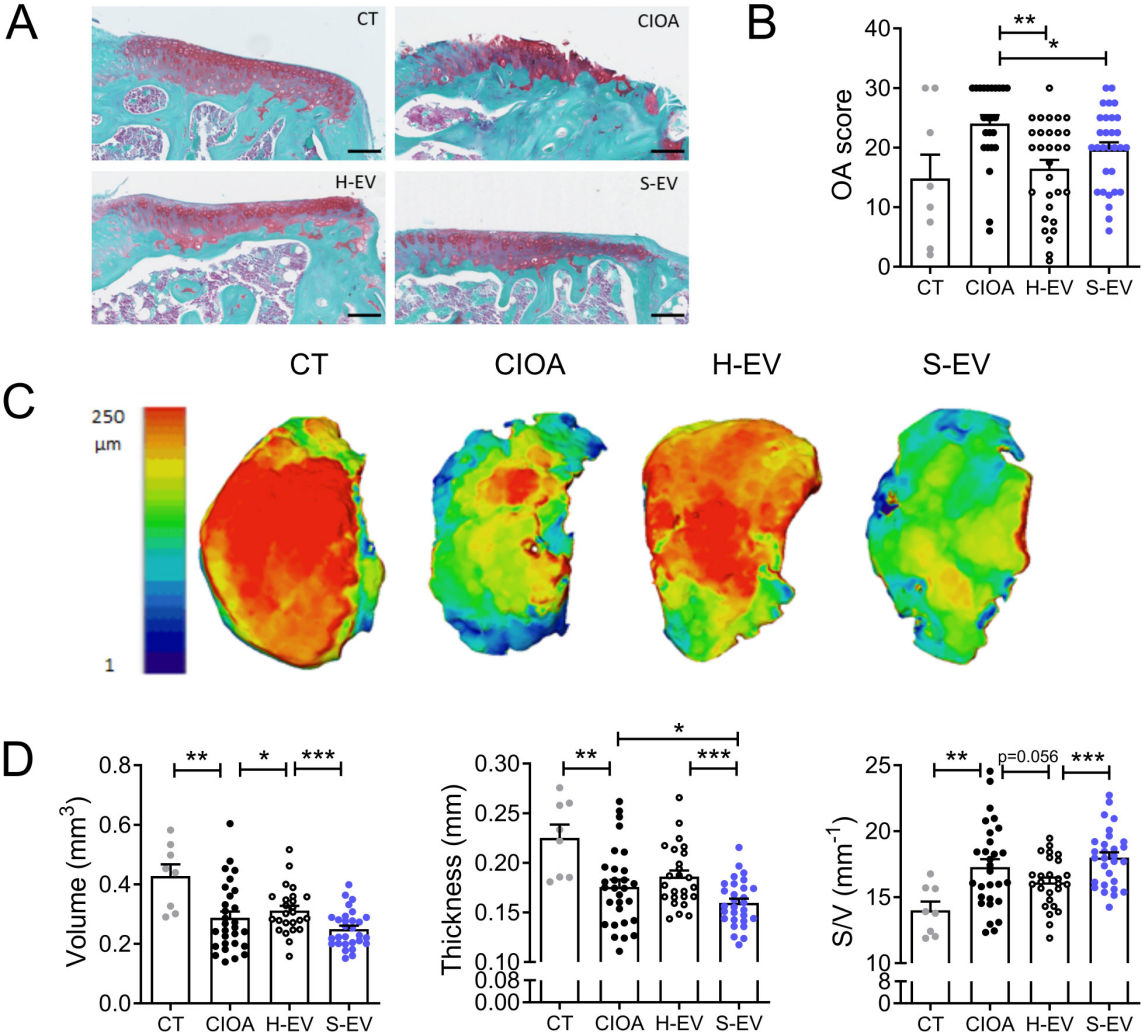
Senescent EVs fail to inhibit cartilage degradation in the collagenase-induced OA model. (**A**) Representative histological sections of tibias from control mice (CT), collagenase-treated mice (CIOA) and CIOA mice that received 250 ng EVs from healthy or senescent ASCs (H-EV or S-EV). Safranin O-Fast green staining. (**B**) Average OA score from histological sections of the different groups of mice. (**C**) Representative 3D reconstructed images of articular cartilage after confocal laser scanning microscopy analysis. (**D**) Histomorphometric parameters (volume, thickness, surface/volume) of 3D images of articular cartilage shown in (**C**). Data are shown as mean ± SEM (*n* = 8–30). Statistical analysis used the Mann-Whitney test for comparing samples pairwise with *p < 0.05, **p < 0.01, ***p < 0.001

To decipher the effect of S-EVs in the joint environment, we compared the impact of S-EVs and H-EVs after intra-articular injection in the CIOA murine model. The OA score, which is higher with the increase of cartilage alterations, was evaluated by histological analysis. The score was lower in the group of mice receiving H-EVs compared to the CIOA group, and similar to that of the healthy control while it was higher in the group of mice receiving S-EVs (Fig. 7A-B). Cartilage degradations were confirmed by CLSM analysis as shown by representative pictures of tibial plateaus from each group of mice (Fig. 7C). The volume of cartilage was higher in H-EVs treated mice compared to CIOA mice while surface degradation, assessed by the surface/volume parameter, tended to be lower (Fig. 7D). Importantly, all the parameters (volume, thickness and surface degradation) were significantly worsened in S-EVs treated mice compared to H-EVs treated mice and not different from CIOA mice. The cartilage thickness was significantly worse after S-EVs treatment compared to the CIOA mice.

**Fig. 8:**
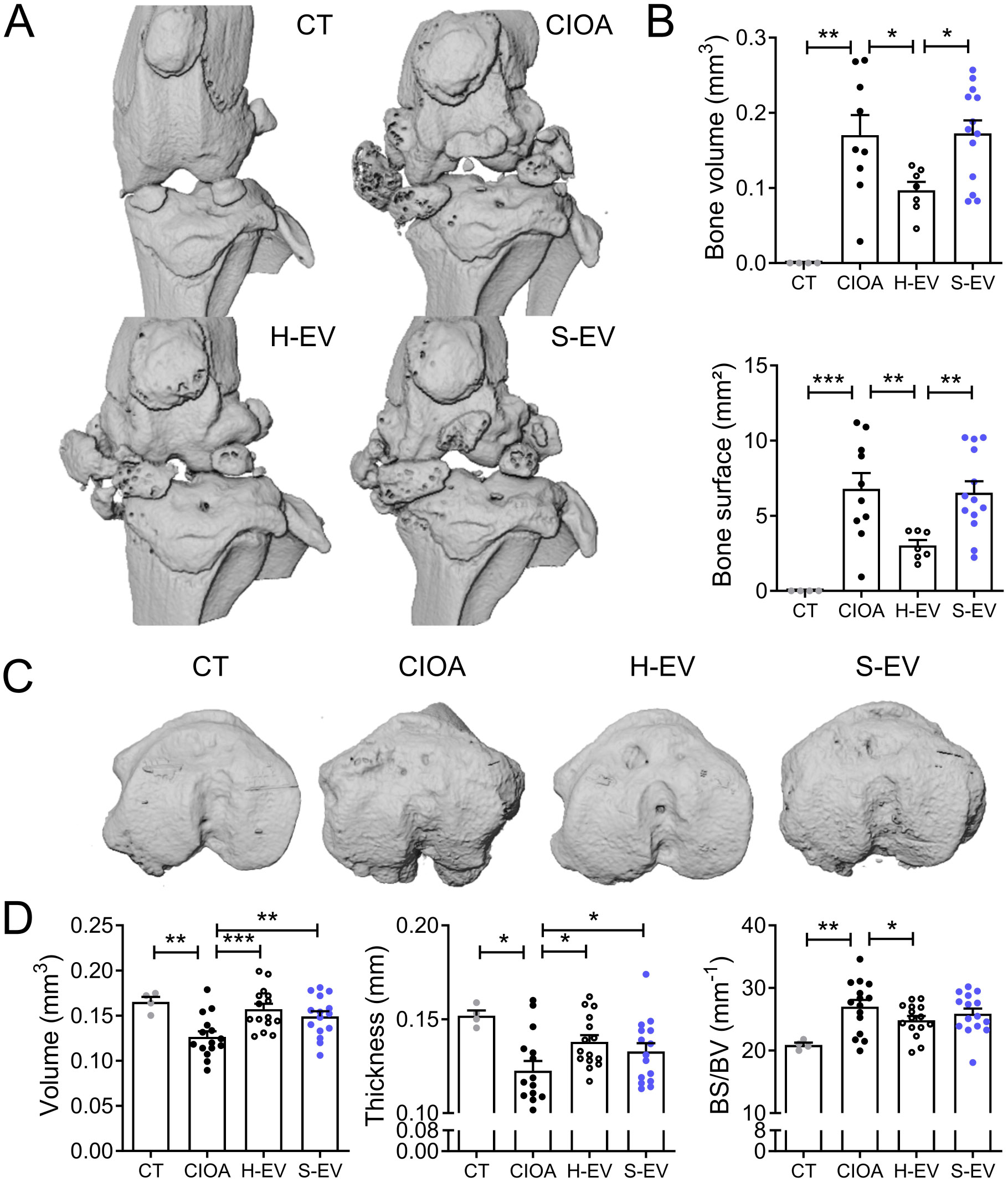
Senescent EVs partly fail to inhibit bone alterations in the collagenase-induced OA model. (**A**) Representative 3D reconstructed images of knee joints from control mice (CT), collagenase-treated mice (CIOA) and CIOA mice that received 250 ng EVs from healthy or senescent ASCs (H-EV or S-EV) after µCT analysis. (**B**) Histomorphometric parameters of calcifications (bone volume and surface) in knee joints of mice are shown in (**A**). (**C**) Representative 3D reconstructed images of sub-chondral bone surface in tibias of the different groups of mice after µCT analysis. (**D**) Histomorphometric parameters (volume, thickness, surface/volume) of subchondral bone in mice shown in (**C**). Data are shown as mean ± SEM (*n* = 4–15). Statistical analysis used the Mann-Whitney test for comparing samples pairwise with *p < 0.05, **p < 0.01, ***p < 0.001

Looking at bone alterations, we observed calcifications in the menisci and the lateral and median ligaments in the CIOA group (Fig. 8A). These calcifications were improved in the group of H-EVs treated mice as quantified by the bone volume and surface parameters (Fig. 8B). By contrast, these parameters were not improved in S-EVs treated mice and were similar to those in the CIOA group. Regarding the sub-chondral bone parameters, the bone volume and thickness were significantly decreased in the CIOA group while the surface degradations were increased (Fig. 8C-D). These parameters were all improved in the group of mice treated with H-EVs. Improvement of these parameters was also noticed in the group treated with S-EVs, but to a lesser extent.

**Fig. 9:**
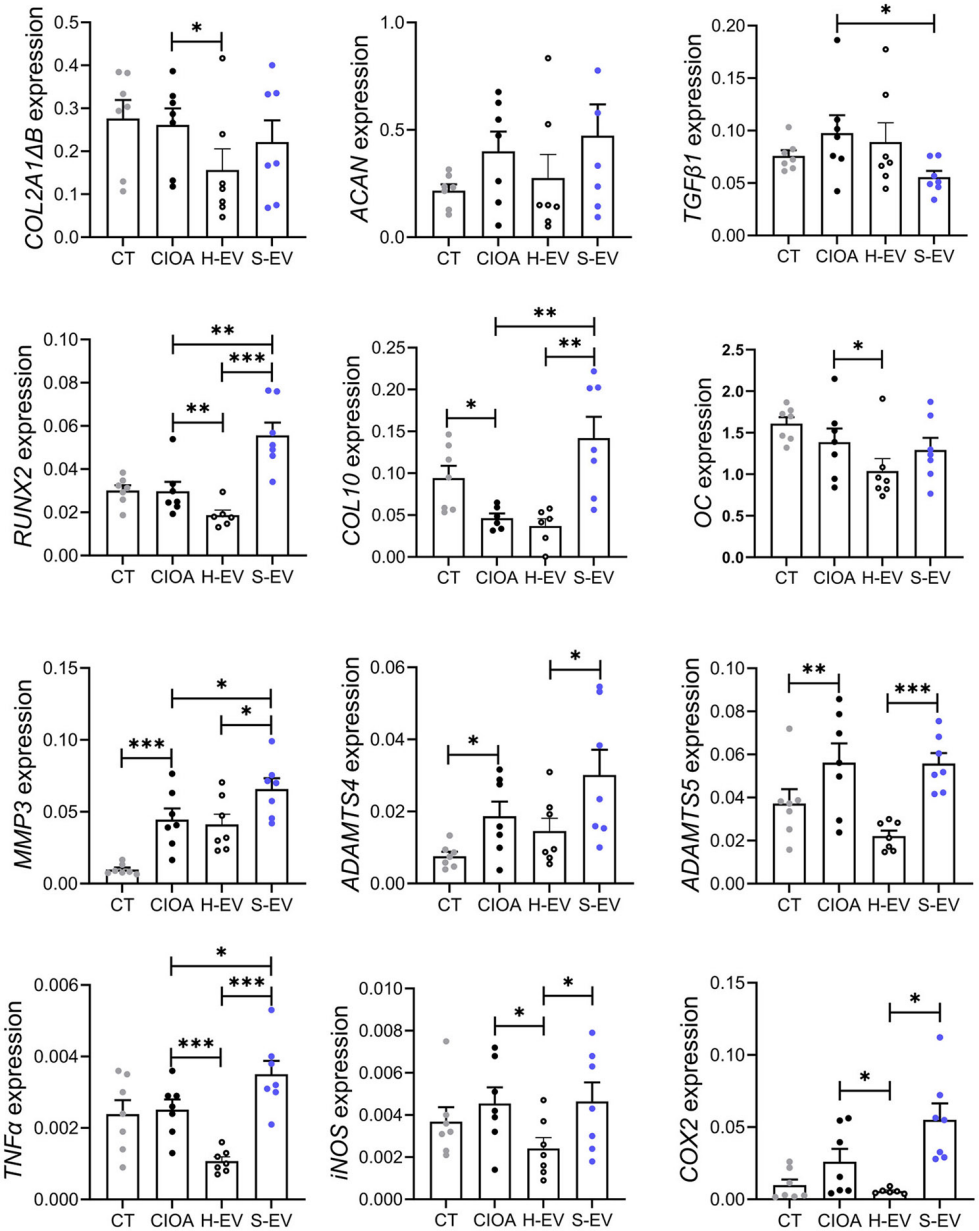
Senescent EVs induce a pro-inflammatory and pro-catabolic phenotype in the joints of collagenase-induced OA mice. Relative expression of several markers related to anabolism, catabolism, and inflammation (*n* = 7/group). Data are shown as mean ± SEM. Statistical analysis used the Mann-Whitney test for comparing samples pairwise with *p < 0.05, **p < 0.01, ***p < 0.001

Finally, we analyzed the expression of several cartilage and bone markers in the joint tissues recovered on day 42. Injection of S-EVs down-regulated the anabolic factor *TGFβ1* and up-regulated the markers of calcification and cartilage hypertrophy (*RUNX2, COL10)*, matrix degradation (*MMP3, ADAMTS4, ADAMTS5*) and inflammation (*TNFα, iNOS, COX2*) compared to CIOA and/or H-EVs treated mice (Fig. 9). Altogether, the analysis of histomorphometric and molecular parameters of articular cartilage, subchondral bone and joint compartment demonstrated a therapeutic effect of H-EVs while S-EVs partly lose this protective function in a relevant murine OA model.

## Discussion

In the present study, we showed that S-EVs exacerbate the catabolic and inflammatory phenotype of OA chondrocytes cultured in proliferative conditions together with the increase of pro-aging factors, notably CDKIs. Furthermore, they aggravate the pro-inflammatory and pro-oxidative phenotype of synovial macrophages and fibroblasts in vitro. As a result, S-EVs cannot prevent the structural alterations in the different joint compartments in vivo, further confirming that contrary to H-EVs, S-EVs activate deleterious signals.

Using ETO, we have generated a model of DNA damage-induced senescence in human ASCs that exhibits all the main features of senescence as soon as day 7 after induction, with high reproducibility. We previously found similar features using bone marrow-derived human MSCs (BM-MSCs) confirming the interest in this chemical inducer of senescence [20]. Here, we showed that S-ASCs produce 3-fold more EVs than H-ASCs but S-EVs contain 2-fold less total RNA. This is in accordance with a previous study using ASCs from young and aged healthy donors reporting slightly higher production of EVs by aging ASCs and similar total protein content in EVs [21]. Another study using BM-MSCs reported that EVs are secreted in higher amounts by cells from old rats than from young rats but contain 3-fold fewer total proteins [22]. The discrepancies cannot be attributed to the process of EV isolation, which was similar in these studies, but likely to the tissue and species of origin of the MSCs used. However, apart from ASCs or BM-MSCs, the current understanding is that the amount of EVs produced by senescent cells is increased and their content is different from that of young cells ([23, 24] and for review, see [25]).

One important finding of this work is that S-EVs partly lose the chondroprotective functions that were observed with H-EVs in vitro. In OA, the therapeutic potential of EVs isolated from healthy or young MSCs has been described in several studies [10, 26, 27]. However, to our knowledge, this is the first study evaluating the effect of EVs isolated from senescent ASCs in OA. We found a similar capacity of S-EVs and H-EVs to improve the anabolic, catabolic and inflammatory markers and, to reduce the percentage of SA-β-Gal positive chondrocytes cultured in vitro in resting conditions. In these conditions, however, S-EVs up-regulated the marker of senescence *p15*. All these markers are indicative of senescence and were up-regulated in OA chondrocytes, which are quiescent in their native state in the cartilage. This was the rationale for investigating the impact of S-EVs in resting conditions although most of the studies used chondrocytes cultured in proliferative conditions [10, 26, 27]. In a single study, umbilical cord MSC-EVs were described to improve the anabolic and hypertrophic markers of chondrocytes cultured in low SVF (1%) [28], which is in line with our findings with H-EVs. By contrast, the catabolic and inflammatory markers and the CDKIs were up-regulated by S-EVs, and not H-EVs, when OA chondrocytes were cultured in proliferative conditions. These results were expected since a loss of function of MSCs and their EVs has been documented when cells are undergoing senescence (for review, see [29]). One interesting finding of this study is that S-EVs exert anti-aging and protective functions on OA chondrocytes uniquely when chondrocytes are cultured in non-proliferative conditions. It is well known that serum-deprived culture conditions induce quiescence, characterized by lack of cellular growth, low metabolism and, downregulation of genes involved in cell division as well as up-regulation of p27 and HES-1. HES-1 expression is sufficient to prevent senescence in naturally quiescent cells, thereby playing a role in stem cell lineages and tissue regeneration [30]. Although we did not find any regulation of HES-1 expression in our settings, further investigation is needed to better understand the underlying mechanisms.

Because chondrocytes are not the single targeted cells of EVs in the joint compartment, we also investigated their impact on synovial macrophages and fibroblasts. The balance between the pro- and anti-inflammatory macrophages was in favour of the M2 phenotype when these cells were treated with H-EVs, while it shifted toward the M1 phenotype with S-EVs treatment. Several M1- and M2-related cytokines were up-regulated by the two types of EVs but the modulation was higher in the presence of S-EVs, suggesting an attempt of synoviocytes to counterbalance the pro-aging signals released by S-EVs. The down-regulation of *TGFβ1* and the up-regulation of *TNFα, NOS2* and *IL6* in macrophages by aged MSC-EVs compared to young MSC-EVs has already been reported [21]. In addition, decreased expression of *SIRT1* in macrophages by senescent EVs has also been found and correlated with the expression of miR-30b-5p [31]. Moreover, our findings indicate that synovial fibroblasts up-regulated the expression of both pro- and anti-catabolic markers activated a pro-oxidative profile upon the addition of S-EVs. Similar to our findings, EVs from senescent fibroblasts up-regulate *MMP1, MMP3* and *IL6* in non-senescent fibroblasts [32, 33].

Altered functions of EVs from aged MSCs have already been reported. Indeed, their immunomodulatory functions or their potential to induce differentiation and proliferation are lower ([21] and for review, see [34]). The pro-angiogenic activity and pro-wound healing effect of oxidative stress-induced senescent MSC-EVs are also decreased compared to EVs from healthy MSCs [35]. In another model of LPS-induced lung injury, aged MSC-EVs were not able to promote M2 macrophage polarization and alleviate inflammation [21]. Accordingly, miRNAs associated with the immune profile of MSCs were shown to be decreased in EVs from aged MSCs, which may be related to the loss of their immunotherapeutic function [36]. Furthermore, the CM of healthy ASCs has been shown to counteract the premature senescence of OA chondrocytes induced by inflammatory stress [37] and we have recently demonstrated the senomorphic function of ASCs-derived H-EVs on chondrocytes (Boulestreau et al., submitted). Our comparative proteomic analysis revealed modulated expression of different proteins in S-EVs. In particular, APOE levels were lower while ICAM-1 and GPC1 were higher compared to H-EVs. These three proteins have been associated with anti-inflammatory functions and the polarization of macrophages toward an M2 phenotype. In S-ASCs, elevated levels of COL15A and LOXL4 were detected. Notably, these proteins exhibit opposing effects, with COL15A being pro-inflammatory and LOXL4 anti-inflammatory [38–40]. Modulated expression of these two proteins has also been associated with senescence [41–43]. The proteins modulated in S-ASCs and S-EVs may have therefore inverse functions, accelerating or decreasing senescence and/or inflammation, suggesting a possible feedback loop to counteract the senescence induction in cells. Interestingly, the role of glutathione metabolism, in particular the role of glutathione-S-transferase mu 2 (GSTM2) has been reported to be associated with a protective effect of young EVs in aging via its anti-oxidant capacity [44]. We found an upregulation of two proteins involved in glutathione metabolism, namely GPX1 and GSTP1, in S-EVs. These two proteins, which are detoxification enzymes might be increased in senescent cells as the result of a protective mechanism to counteract the oxidative stress associated with senescence. Their role has not been investigated in the present study and in the context of OA but warrants to be done in the future. Nevertheless, mass spectrometry analysis likely detects only the most abundant proteins, potentially leading to an incomplete representation of the protein cargo and thus impeding the comprehensive assessment of EVs’ overall function. Furthermore, it cannot be excluded that the miRNA components of the EV cargo may be also important players in the EV functions, and this has not been investigated in the present study. Here, the functional assays suggested an overall pro-aging effect of S-EVs on the various cell types of the joint compartment that has been confirmed in vivo using a murine model of inflammatory OA in which the therapeutic effect of H-EVs was not observed with S-EVs.

In conclusion, we provide evidence that S-EVs do not exert the chondroprotective effect observed with H-EVs both in vitro, on OA chondrocytes and synoviocytes, and in vivo, in a murine model of OA. Further studies are required to unravel the mechanisms of action of H-EVs that are lost in S-EVs. This could help to identify factors that could be loaded in engineered EVs for higher effectiveness and design future clinical trials for the treatment of OA.

### Electronic supplementary material

Below is the link to the electronic supplementary material.

Supplementary Material 1

## Data Availability

No datasets were generated or analysed during the current study.
