# Correction: Extracellular vesicles from senescent mesenchymal stromal cells are defective and cannot prevent osteoarthritis

**DOI:** 10.1186/s12951-024-02655-6

**Published:** 2024-07-16

**Authors:** Jérémy Boulestreau, Marie Maumus, Giuliana M. Bertolino, Karine Toupet, Christian Jorgensen, Danièle Noël

**Affiliations:** 1 IRMB, University of Montpellier, INSERM U1183, Hôpital Saint-Eloi, 80 Avenue Augustin Fliche, Montpellier Cedex 5, 34295 France; 2 Clinical Immunology and Osteoarticular Disease Therapeutic Unit, Department of Rheumatology, CHU Montpellier, Montpellier, France

**Correction:** ***Journal of Nanobiotechnology*** **(2024) 22:18**

10.1186/s12951-024-02509-1.

In the original version of this article, the given and family names of all authors were incorrectly structured as: Boulestreau Jérémy, Maumus Marie, Bertolino M. Giuliana, Toupet Karine, Jorgensen Christian and Noël Danièle. The correctly structured given and family names of all authors are: Jérémy Boulestreau, Marie Maumus, Giuliana M. Bertolino, Karine Toupet, Christian Jorgensen and Danièle Noël. The original article has been corrected.

